# An innovative temperature‐controlling handpiece for face and body skin laxity and tightening treatment with radiofrequency

**DOI:** 10.1111/srt.13385

**Published:** 2023-06-06

**Authors:** Pacifici Alvaro, Oddo Alberto, Fedeli Matia, Pennati Beatrice Marina

**Affiliations:** ^1^ Clinica Laser Perugia Perugia Italy; ^2^ Clinical Research & Practice El.En. Group Calenzano Italy

**Keywords:** collagen, esthetics, Radiofrequency therapy, skin, skin temperature, women

Dear Editor,

In this study, a temperature‐controlled radio frequency (RF) was used to improve tightening and skin laxity in different body areas using an innovative temperature‐controlling handpiece. Despite the many choices in the scenario against signs of aging, many patients may remain unsatisfied due to medications or treatments requiring months to be effective. This affects adherence to regimens and, therefore, compliance with treatment. In recent years, lasers, therapeutic ultrasounds, and different energy sources have been investigated for their possible role as a solution for tightening and skin laxity. Within these, there is medical RF, a form of electromagnetic energy generating heat proportionally to the electrical resistance of the tissue when applied to skin tissues. RF lays its foundations in the early 20th century as a solution for back pain and neuralgias and later on heating of the dermal tissues was considered for skin rejuvenation. Indeed, surgery has extensively used this heat source for example for homeostasis and tissue ablation (so‐called electro‐surgery) but, it has been recently employed for shrinking of lax connective tissues thanks to its collagen denaturation properties [1]. As a matter of fact, fibroblasts produce collagen molecules synthesizing three chains of polypeptides wrapped around one another in a triple helix. This is the first structure involved during the denaturation phenomenon of thermal shrinkage. Indeed, heat destroys all the collagen heat‐labile intramolecular cross‐links leading to the protein “unwinding” and change in structure from a highly ordered crystalline form to a randomly distributed and gel‐like shape [2]. Fibroblasts are also implicated in neocollagenesis and consequent tissue remodeling, contributing to the desired cosmetic result when heated. The specific connective tissue heat‐induced behavior and shrinkage extent depend on several factors including the maximum exposure temperature and time, tissue hydration, and age.

RF energy can be applied to tissue up to six points on the tip of a probe. In general, the more poles are used, the less current is required to achieve the same effect because the current passes through a much smaller volume of tissue. Moreover, an active cooling mechanism is required when a monopolar RF is used because the electrode touching the skin has to be cooled down to preserve the epidermis from thermal damage [3]. For this reason, a multipolar handpiece is typically used in most recent devices for face and body treatments. Different dimensions are available based on the patient characteristics and endpoint wanted [4]. For example, in the tripolar handpiece, one tip is acting as a positive pole while the others are a negative one. The current flowing through the positive pole will be double the one singularly flowing through each negative pole. To avoid the positive pole and tissue overheating, each electrode is turned into acting as the common pole by applying a sequence of electrical modulation. This way, the device requires no active cooling of the electrodes or the skin. The Subject Device (Setis, Luxea, Deka M.E.L.A., Calenzano, Italy) delivers RF energy at a frequency of 1 MHz and a maximum power of 50 W. Five applicators of different sizes with a cutaneous temperature sensor are available (three tips: Very Small, Small, Medium, Large; six tips: Hexapolar) for the treatment of different depths and anatomical sites including the face (i.e., perioral, periocular, cheek), arms, neck, buttocks, abdomen, and thighs. The system is indicated for treating skin texture, skin laxity, body contouring, and cellulite. Apparently, 40–48°C is the ideal epidermal surface temperature since this correlates with a dermal temperature of 70°C required for the denaturation of collagen [5]. For this reason, after defining the optimal treatment temperature of cutaneous heating (from 40 to 45°C), thanks to the continuous feedback from the software, the system stops and starts over the energy supply automatically never overcoming the pre‐set temperature threshold. This way, every overheating is spared and so the unwanted side effects. Thus, the handpiece performance is maximized in every session. The heating is more homogeneous on the interested body area.

In this study, 40 patients were considered. They were women with a mean age of 60 (± 5). 70% (28) of them underwent 4 treatment sessions on the face with the tripolar handpiece for the face area (15 min, up to 35 W, 41°C) while 30% (12) of subjects were treated with the hexapolar handpiece on the abdomen (15 min, up to 50 W, 45°C). Treatment side effects such as erythema, the sensation of heat sensation, and pain in the treatment area were monitored. None was observed during the treatment or the 4‐8‐12 weeks’ follow‐up time points. A 4‐point Global Aesthetic Improvement Scale (GAIS) (None‐0, Slight‐1, Mild‐2, and Excellent‐3) was used to assess the improvement patient's skin tone before and 4‐8‐12 weeks after the last treatment. As shown in Figure 1, after 4 weeks from the last treatment, 52% of patients showed a GAIS score of 2 and 48% of 3; after 8 weeks, 88% of the patients still exhibit good results (GAIS score = 2, 55%, score 3 = 33%); finally, after 12 weeks, 73% of the subjects’ presented results with GAIS score 2 (50%) and score 3 (23%) so far. An example of clinical treatment results in reported in Figure 2.

**FIGURE 1:**
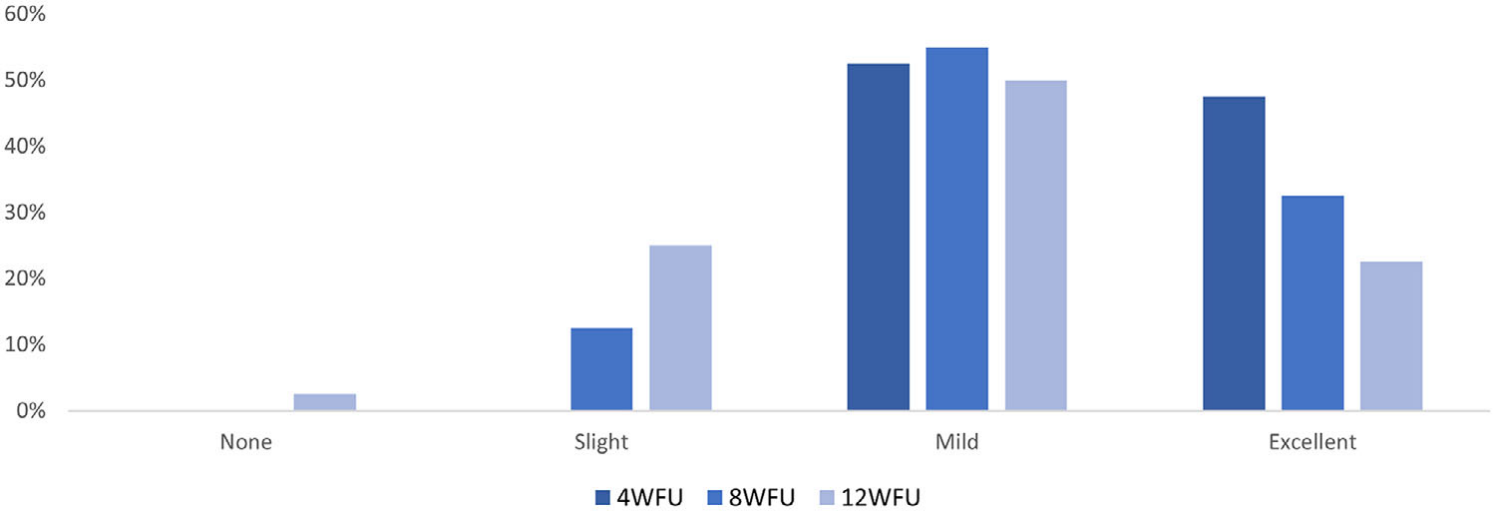
A 4‐point Global Aesthetic Improvement Scale (GAIS) (None, Slight, Mild, and Excellent) was used to assess the improvement patient's skin tone before and 4‐8‐12 weeks after the last treatment. The majority of patients showed mild to excellent results even after 8 or 12 weeks from the last treatment. FU = Follow‐Up.

**FIGURE 2:**
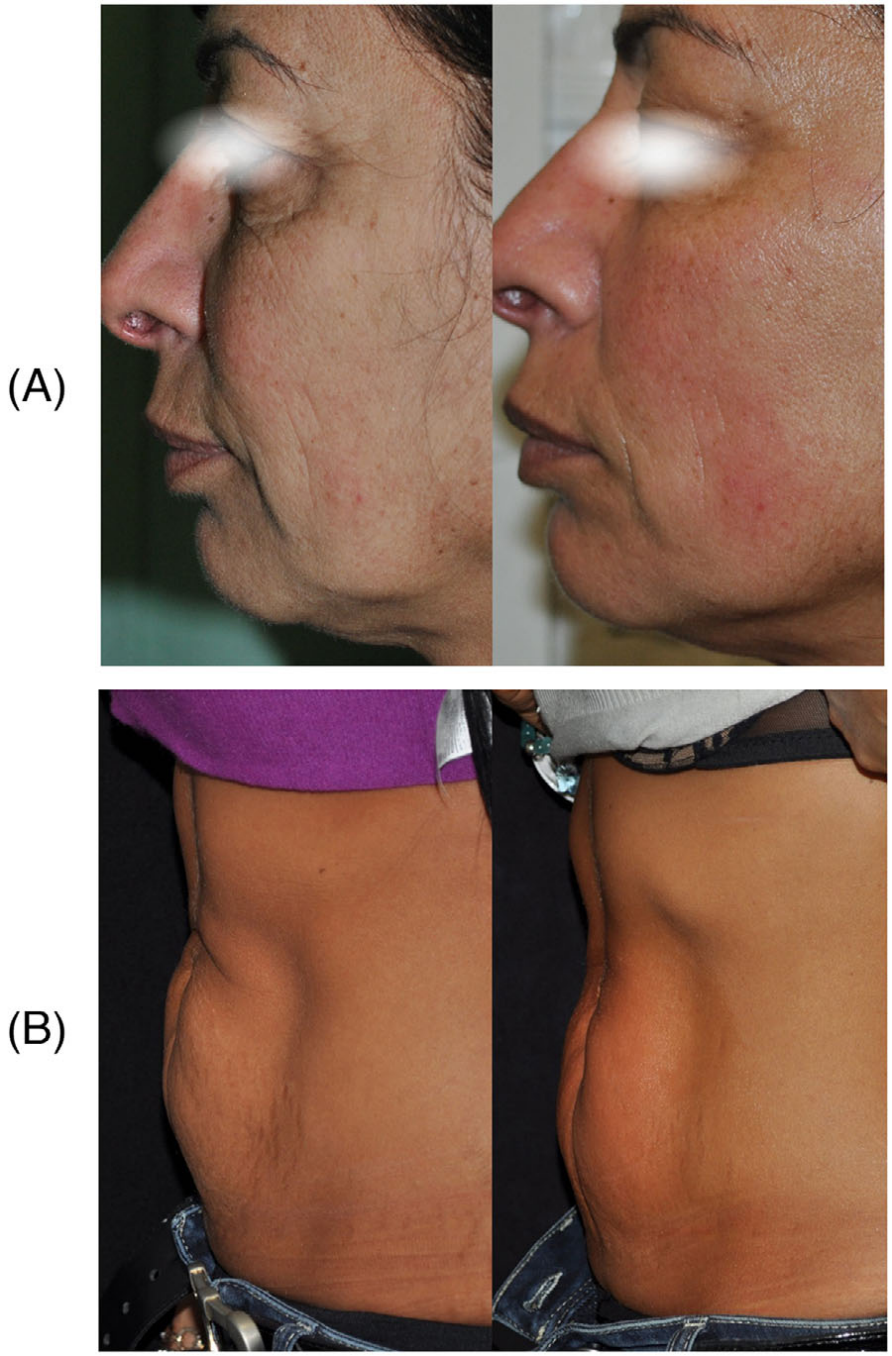
Clinical treatment results after the first session with the study device. (A) Twenty‐eight subjects (70%) underwent four treatment sessions on the face with the tripolar handpiece (15 min, up to 35 W, 41°C). (B) Twelve patients (30%) were treated with hexapolar handpieces on the abdomen (15 min, up to 50 W, 45°C). Both types of treatment showed mild to excellent results even after 12 weeks from the last treatment session.

Our study has shown great skin tone and texture improvement in the patient's cheek area thanks to RF action on collagen. Overall, the treatment was well tolerated and none of the patients showed discomfort. Indeed, in this study, we demonstrated that constantly monitoring the temperature during RF treatment is highly innovative and crucial for achieving better results and minimizing side effects. This means having faster, operator‐independent, and easy‐manageable technology compared to standards. As a support, there are a lot of scientific data about the efficacy of using a multipolar handpiece [4] for face and body tightening and skin laxity but none have feedback about superficial body temperature in real‐time [6]. Therefore, constant scientific research for updating and improving the available devices is needed.

In conclusion, thanks to RF action on collagen, our study has interestingly shown great skin tone and texture improvement on the patient's face and abdomen. Therefore, the new handpiece characteristic presented within the non‐invasive and operator‐independent RF system could be considered a safe, quick, and effective procedure for patients’ face and body skin laxity and tightening even in subjects with a considerable percentage of lax skin tissue.

## CONFLICT OF INTEREST STATEMENT

Pennati Beatrice Marina is employed at El.En. Group. The other authors declare no conflict of interest.

## Data Availability

None.

## References

[1] Levenberg A . Clinical experience with a TriPollar radiofrequency system for facial and body aesthetic treatments. Eur J Dermatol. 2010;20(5):615‐619. 10.1684/EJD.2010.1042 20627852

[2] Fujii KK , Taga Y , Takagi YK , Masuda R , Hattori S , Koide T . The thermal stability of the collagen triple helix is tuned according to the environmental temperature. Int J Mol Sci. 2022;23(4):2040. 10.3390/IJMS23042040 35216155 PMC8877210

[3] Yu JNT , Huang P . Use of a TriPollar radio‐frequency device for the treatment of Acne Vulgaris. J Cosmet Laser Ther. 2011;13 (2):50‐53. 10.3109/14764172.2011.564626 21401377

[4] Sadick NS , Nassar AH , Dorizas AS , Alexiades‐Armenakas M . Bipolar and multipolar radiofrequency. Dermatol Surg. 2014;40:S174‐S179. 10.1097/DSS.0000000000000201 25417571

[5] Alam M , Hsu TS , Dover JS , Wrone DA , Arndt KA . Nonablative laser and light treatments: Histology and tissue effects–A review. Lasers Surg Med. 2003;33(1):30‐39. 10.1002/LSM.10195 12866119

[6] de la Torre J.R , Moreno‐Moraga J , Muñoz E , Navarro PC . Multisource, phase‐controlled radiofrequency for treatment of skin laxity: Correlation between clinical and in‐vivo confocal microscopy results and real‐time thermal changes. J Clin Aesthet Dermatol. 2011;4(1)28‐35. PMC3030214 21278896

